# Exploring the In Vitro Antibacterial Potential of Specific Probiotic Strains against Oral Pathogens

**DOI:** 10.3390/microorganisms12030441

**Published:** 2024-02-21

**Authors:** Diletta F. Squarzanti, Federica Dell’Atti, Alessandro C. Scalia, Ziba Najmi, Andrea Cochis, Patrizia Malfa

**Affiliations:** 1 Synbalance Srl, 21040 Origgio, VA, Italy; p.malfa@synbalance.care; 2 Immunomics Laboratory, Department of Health Sciences, Center for Translational Research on Autoimmune and Allergic Diseases (CAAD), University of Eastern Piedmont, 28100 Novara, NO, Italy; federica.dellatti@uniupo.it; 3 Biomedical Materials Laboratory, Department of Health Sciences, Center for Translational Research on Autoimmune and Allergic Diseases (CAAD), University of Eastern Piedmont, 28100 Novara, NO, Italy; alessandro.scalia@uniupo.it (A.C.S.); ziba.najmi@uniupo.it (Z.N.); andrea.cochis@uniupo.it (A.C.)

**Keywords:** probiotics, oral, pathogens, infection, host interaction, microbiota, dysbiosis

## Abstract

The microbiota in the oral cavity has a strict connection to its host. Its imbalance may determine oral diseases and can also have an impact on the systemic health. Probiotic strains may help in the restoration of a balanced condition. For this purpose, we screened the antibacterial and antiadhesive activities of many viable probiotic strains (*Lactobacillus acidophilus* PBS066, *Lactobacillus crispatus* LCR030, *Lactobacillus gasseri* LG050, *Lactiplantibacillus plantarum* PBS067, *Limosilactobacillus reuteri* PBS072, *Lacticaseibacillus rhamnosus* LRH020, *Bifidobacterium animalis* subsp. *lactis* BL050, *Lacticaseibacillus paracasei* LPC 1101, *L. paracasei* LPC 1082, and *L. paracasei* LPC 1114) against two main oral pathogens, *Streptococcus mutans* and *Aggregatibacter actinomycetemcomitans*, involved in dental caries and periodontal disease development and progression. Considering both the agar overlay preventive and treatment models, seven probiotics determined greater inhibition zones against the tested pathogens. This behavior was further analyzed by the plate count method and scanning electron microscope imaging. *L. plantarum* PBS067, *L. rhamnosus* LRH020, *L. paracasei* LPC 1101, *L. paracasei* LPC 1082, and *L. paracasei* LPC 1114 prevent the growth and adhesion of oral pathogens in a strain-specific manner (*p* < 0.0001). These probiotics might be considered as an alternative effective adjuvant to improve oral and systemic well-being for future personalized treatments.

## 1. Introduction

The oral cavity hosts its characteristic microbiota, which comprises more than 700 different kinds of microorganisms, spread out in specific niches including the tongue, cheeks, hard and soft palates, saliva, teeth, throat, pharynx, and gingival sulcus [1,2,3]. In the 1680s, Antony van Leeuwenhoek studied the oral microbiota, calling it as “little living animalcules prettily moving”. Nowadays, thanks to sophisticated biotechnological techniques, the human oral microbiome is still studied, resulting it being the second most complex in terms of species after the gut microbiome [2]. The interaction between the oral microbiota and the host is complex, since the former creates a polymicrobial biofilm that is strongly attached to oral surfaces and comprehends commensal and pathogenic bacteria and other microorganisms such as yeasts, fungi, and viruses [1,4]. In particular, bacterial communities in the mouth mainly belong to six major phyla (Firmicutes, Bacteroidetes, Proteobacteria, Actinobacteria, Spirochaetes, and Fusobacteria) [3].

In the presence of risk factors, such as an unbalanced diet, alcohol consumption, smoke, improper hygiene practices, or antibiotic use, the microbial community can shift to a dysbiotic state, in which an increase in the number of pathogenic bacteria and the production of virulence factors occur [5]. The dysbiosis of the microbiota is linked to the onset or progression of many oral diseases, such as dental caries, periodontitis, recurrent aphthous stomatitis, and even tumors, as well as extra-oral pathologies, such as diabetes and cardiovascular diseases [3,6,7]. Among the oral bacteria, *Streptococcus mutans* and *Aggregatibacter actinomycetemcomitans* can become opportunistic pathogens in dysbiosis-promoting conditions and behave as pathobionts.

Oral streptococci, including *S. mutans*, are linked to pyogenic and non-pyogenic infections in the mouth and other anatomical districts. *S. mutans* is a Gram-positive facultative anaerobe, and it is considered the major contributor to dental caries and teeth decay due to its acidogenic and aciduric properties, in addition to its capacity to synthesize large amounts of extracellular polymers composed of glucan [8,9,10,11]. It forms a biofilm on tooth surfaces—the so-called dental plaque, one of the most common diseases worldwide. It has been also associated with extraoral pathologies, such as infective endocarditis or atherosclerosis [9,12,13].

HACEK microorganisms are Gram-negative bacteria naturally resident in the oropharyngeal mucosa, comprising *Haemophilus* spp., *Aggregatibacter actinomycetemcomitans*, *Cardiobacterium hominis*, *Eikenella corrodens*, and *Kingella kingae*. The uncontrolled proliferation of these bacteria can lead to a broad range of infections, with infective endocarditis being a notable manifestation [14]. Among them, *A. actinomycetemcomitans* is a facultative anaerobe, oral commensal bacterium, which can behave as an opportunistic pathobiont [15,16]. In predisposing conditions, it can determine an excessive inflammatory response that leads to aggressive periodontitis, avoiding the correct periodontal tissue remodeling [17]. Additionally, certain serotypes possess cytokine-binding molecules able to influence the host’s immune system [18]. Moreover, some *A. actinomycetemcomitans* strains have been associated with the risk of coronary artery disease development [19].

Periodontal pathogens modulate the immune response causing imbalances not only in the site they are colonizing but also at a systemic level. Indeed, periodontal infections have been linked to several pathologies in distant organs, such as gastrointestinal and colorectal cancers, diabetes, insulin resistance, and cardiovascular diseases, and the increased risk of development of non-alcoholic fatty liver disease, type 2 diabetes, and atherosclerotic vascular diseases [9,20,21,22,23,24,25].

The standard treatment for chronic oral diseases is non-surgical periodontal therapy, including oral hygiene procedures, scaling, and root planing. In such cases, the use of antiseptics (e.g., chlorhexidine) or antibiotics as supplemental therapy can be helpful if the standard treatment alone is not effective [26]. Nevertheless, the constant use of antiseptics and antibiotics increases the resistance of oral pathogens to these substances or results in dysbiosis, promoting the proliferation of pathogenic/opportunistic bacteria [27]. The issue of antibiotic resistance has been increasing over the years, thus leading to the urgent need for new types of alternative treatments.

Lactic acid bacteria (LAB) belong to Gram-positive, catalase-negative, non-spore-forming cocci or rods; they tolerate very low pH levels. The LAB demonstrating health benefits, safety traits, and the ability to colonize different anatomical niches may be used as probiotics to sustain the gut microbiota. They can be found in fermented foods, such as yoghurt and dairy products, or in functional foods, such as drinks, food supplements, or drugs. As defined by the Food and Agriculture Organization (FAO) and the World Health Organization (WHO), probiotics are “live microorganisms which when administered in adequate amounts confer a health benefit on the host” [28].

Probiotic-based adjuvant therapies may be useful for treating or even preventing these chronic infections and restoring the oral microbiota, achieving benefits for oral health and the overall well-being [26,29,30,31,32]. The major goal of probiotic treatment is to enhance the presence of beneficial commensal bacteria, which might be able to trigger an anti-inflammatory response as well as an antibacterial and antiadhesive effect against pathogenic microbes [33,34,35,36]. Specific probiotic supernatants have been demonstrated to reduce biofilm formation and virulence factor expression of putative opportunistic oral pathogens [37,38,39].

In this scenario, the present study aims to set up a preliminary in vitro screening to investigate the antimicrobial and antiadhesive properties of specific probiotic strains against two main pathogens of the oral cavity, *S. mutans* and *A. actinomycetemcomitans*. They are both facultative anaerobes and are representatives of Gram-positive and Gram-negative bacteria, respectively. In particular, *A. actinomycetemcomitans* is commonly used as a model microorganism in the in vitro study of aggressive periodontitis and the positive effect of probiotics in this context [36,37,38,39]. It can be considered a key microorganism not only in oral but also in systemic infections [19,40,41,42,43,44]. This research work aims at shedding light on the improvement of the oral cavity well-being by reducing its opportunistic pathogens. So, the final goal of this project is to exploit the probiotic biotherapeutic potential in human health to reach future personalized approaches.

## 2. Materials and Methods

### 2.1. Bacterial Strain Cultures

#### 2.1.1. Probiotic Strains

*Lactobacillus* and *Bifidobacterium* strains used in this study are deposited in the German or Belgian cell culture collections of microorganisms (DSMZ and LMG, respectively). *Lactobacillus acidophilus* PBS066 (DSM 24936), *Lactobacillus crispatus* LCR030 (LMG P-31003), *Lactobacillus gasseri* LG050 (LMG P-29638), *Lactiplantibacillus plantarum* PBS067 (DSM 24937), *Limosilactobacillus reuteri* PBS072 (DSM 25175), *Lacticaseibacillus rhamnosus* LRH020 (DSM 25568), *Bifidobacterium animalis* subsp. lactis BL050 (DSM 25566), *Lacticaseibacillus paracasei* LPC 1101 (DSM 34558), *Lacticaseibacillus paracasei* LPC 1082 (DSM 34557), and *Lacticaseibacillus paracasei* LPC 1114 (DSM 34559) were cultured overnight at 37 ± 1 °C in De Man, Rogosa and Sharpe (MRS) broth (VWR International Srl, Milan, Italy). All the lactic acid bacteria were kindly provided by Synbalance Srl (Origgio, Varese, Italy).

#### 2.1.2. Pathogen Strains

*Streptococcus mutans* (purchased from American type culture collection, Manassas, VA, USA; ATCC 700610, Gram-positive) and *Aggregatibacter actinomycetemcomitans* (DSM 11123, Gram-negative) were chosen as representative oral pathogens. The pathogens were cultured overnight at 37 °C in brain heart infusion and tryptic soy broth (BHI and TSB, both from Biolife Italia Srl, Milan, Italy), respectively.

All the bacterial strains were freshly renewed before each experiment.

### 2.2. Screening of Probiotic Antibacterial Effect by Agar Overlay Assay

The overlay assay was performed on agar plates, where a probiotic strain with potential bacteriocin production was drop-spotted and then overlaid on the top with a layer of soft agar containing a pathogen strain to be tested for bacteriocin sensitivity [45,46].

#### 2.2.1. Preventive Experiments

This set of experiments aimed to determine the possible effect of the probiotic strains in the inhibition of the pathogen growth when applied before the infection.

Briefly, 10 μL of each probiotic strain was spotted on the surface of an MRS agar plate, and the drops were allowed to dry under a laminar flow hood. Plates were incubated for 48 h (h) at 37 ± 1 °C in anaerobic conditions by using Anaerocult A (Millipore, distributed by VWR International Srl) to allow the development of colonies. Then, 10 μL of *S. mutans* or *A. actinomycetemcomitans* was inoculated in 10 mL of the proper melted soft agar medium (BHI or TSB, respectively, containing 7.5 g/L of agar) and poured on top of the MRS agar plates spotted with probiotics. The plates were incubated at 37 °C ± 1 for 24 h.

#### 2.2.2. Treatment Experiments

This set of experiments differed from the previous one since the probiotics were seeded in the moment of the establishment of the infection, so both probiotics and pathogens were put together almost simultaneously.

Briefly, 10 μL of each probiotic was drop-spotted on an MRS agar plate. Immediately after the drops dried, the pathogens were inoculated in the proper soft agar medium and poured as mentioned before and at the same concentration to overlay the probiotics spotted onto the MRS plates. All the plates were then incubated at 37 ± 1 °C for 24 h.

Since the LAB metabolism induces an acidification of the medium, pristine MRS broth at pH 4.5 was used as the control to exclude the potential effect of the acidic pH on *S. mutans* and *A. actinomycetemcomitans* growth.

Inhibitory properties of the probiotic strains against the pathogenic bacteria were evaluated by measuring the diameter of the inhibition halos after incubation. Each zone was measured in different directions to ensure that the diameter measurements were representative. All the experiments were performed in triplicate and repeated 3 times independently.

### 2.3. Colony Forming Unit (CFU) Count of Adherent Cells in the Preventive Model and Image Acquisition

To further investigate and confirm the results obtained in the preventive model of the agar overlay assay, in which the LAB performance was demonstrated to be better than in the treatment one, probiotic strains with the stronger effect against both the pathogens tested were analysed also by using CFU.

Briefly, 50 μL of the overnight probiotic cultures of *L. crispatus* LCR030, *L. gasseri* LG050, *L. plantarum* PBS067, *L. rhamnosus* LRH020, *L. paracasei* LPC 1101, *L. paracasei* LPC 1082, and *L. paracasei* LPC 1114 (≈10^9^ colony forming unit/mL, CFU/mL) was drop-spotted in the centre of sterile glass coverslips (12 mm diameter, Waldemar Knittel Glasbearbeitungs GmbH, Braunschweig, Germany) in a 24-multi-well plate (Biosigma, Cona, Venice, Italy) and incubated at 37 °C for 90 min (min) to allow them to adhere to the surface [47]. A drop of the pristine MRS broth was used as the control. The remaining drops containing non-adherent cells were removed and 50 μL of each pathogen, adjusted in sterile phosphate buffer saline 1X (PBS, VWR International Srl, Milan, Italy) at a concentration of 10^6^ CFU/mL, was drop-spotted on top of the probiotic adherent cells. The multi-well plates were then incubated for 24 h at 37 ± 1 °C.

To perform CFU counts of adherent cells, the coverslips were sonicated (Sonica 3200MH S3, Soltec Srl, Milan, Italy) in 1 mL of sterile PBS 1X to mechanically detach all the adherent bacteria. Several serial dilutions were performed and plated on a homofermentative–heterofermentative differential (HHD, Biolife Italiana Srl, Monza, Italy) agar medium to discriminate the pathogen from the probiotic cells, thanks to their different morphology. Since HHD is a preferred medium for LAB but not for other genera, we tested the ability of the two pathogens tested to grow in this condition and to form colonies in a comparable amount with respect to their preferred medium. The plates were incubated for 24–48 h at 37 ± 1 °C and the pathogen colonies were counted. All the experiments were performed in triplicate and repeated 3 times independently.

Finally, the morphology and presence of the bacterial cells were investigated by scanning electron microscopy. Briefly, the coverslips prepared as previously described were fixed with 2.5% glutaraldehyde (Electron Microscopy Sciences, Hatfield, PA, USA) and left overnight at 4 °C. Then, to allow complete dehydration of the samples, an ethanol scale (from 70 to 100%, 1 h each; Merck, Italy) was performed. Subsequently, hexamethyldisilazane (Electron Microscopy Sciences, Hatfield, PA, USA) was added for 20 min to prevent the collapse of the bacterial cell structure. Samples were then coated with a thin layer of gold using a sputter coater machine (DII-29030SCTR Smart Coater, JEOL SpA, Basiglio, Milan, Italy). The images were acquired by using a bench scanning electron microscope (SEM, JSM-IT500, JEOL SpA) at different magnifications (5000× and 10,000×).

### 2.4. Statistical Analysis

Ordinary one-way ANOVA followed by Dunnett’s multiple comparison test was performed using the GraphPad Prism version 8.0.2 for Windows (GraphPad Software, San Diego, CA, USA, www.graphpad.com accessed on 10 January 2024). Results were expressed as mean ± standard deviation (SD). Statistical significance was fixed at *p* < 0.05.

## 3. Results

### 3.1. Antibacterial Properties of Probiotic Strains

Ten probiotic strains were first screened for their antibacterial properties against *S. mutans* and *A. actinomycetemcomitans* by using an agar overlay assay. The inhibition halos were measured after 24 h of incubation. The results of the treatment and preventive models were compared to evaluate whether the antibacterial effect could be exerted only if the antimicrobial compounds accumulate in the environment together with the colony formation or if they could be effective also at the time of the infection establishment.

**Figure 1:**
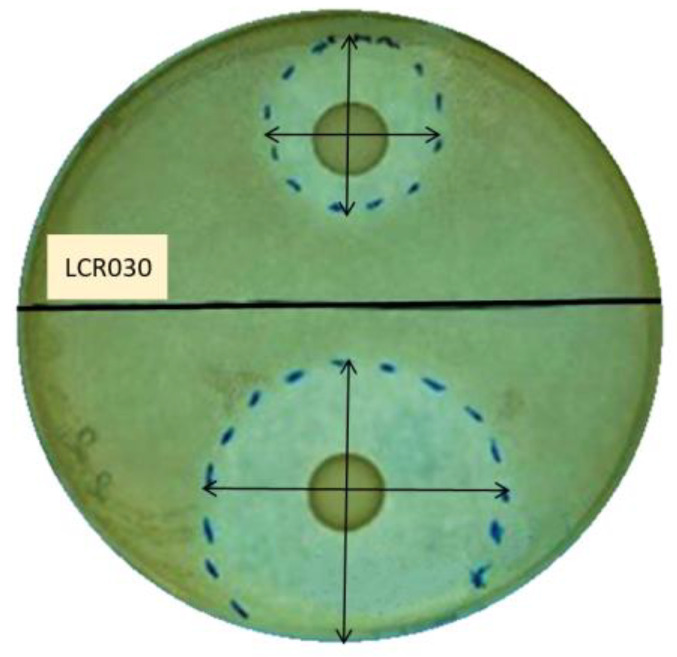
Representative image of inhibition halos. *L. crispatus* LCR030 antibacterial activity against *A. actinomycetemcomitans* in the preventive (bottom part of the plate) and treatment (top of the plate) models. The halo areas were indicated by dashed blue lines and the diameters were measured; the results were expressed in mm.

All the probiotic strains tested were able to counteract the pathogen growth in both the preventive and treatment experiments. As expected, the preventive model results demonstrated a greater inhibition of pathogens with respect to the treatment one, except for *L. reuteri* PBS072, which did not improve the inhibition zone in the preventive model (Table 1; Figure 1).

In the preventive model, *L. crispatus* LCR030 and *L. paracasei* LPC 1101 produced halos with diameters > 45 mm, showing the strongest effect against *S. mutans*; while against *A. actinomycetemcomitans*, the same result was achieved by *L. gasseri* LG050, *L. plantarum* PBS067, and *L. paracasei* LPC 1114 (Table 1).

In the treatment assays, many probiotic strains were active against *S. mutans* in an intermediate way, showing diameter halos ranging from 16 to 30 mm, with the exception of *L. gasseri* LG050 and *B. lactis* BL050, which had lower effects. The best probiotic performers against *A. actinomycetemcomitans* were instead *L. gasseri* LG050 and *L. plantarum* PBS067, showing diameter halos ranging from 31 to 45 mm (Table 1).

Considering all the conditions, the strains that showed the strongest inhibition against the tested pathogens in both models were *L. crispatus* LCR030, *L. gasseri* LG050, *L. plantarum* PBS067, *L. rhamnosus* LRH020, *L. paracasei* LPC 1101, *L. paracasei* LPC 1082, and *L. paracasei* LPC 1114.

### 3.2. Inhibition of Pathogen Adhesion and Growth by Probiotic Strains

The agar overlay assay revealed better performances of the LAB in the preventive experiments rather than in the treatment model, so we deepened this aspect in the following assay. Seven out of ten probiotic strains that determined the greater inhibition zones (*L. crispatus* LCR030, *L. gasseri* LG050, *L. plantarum* PBS067, *L. rhamnosus* LRH020, *L. paracasei* LPC 1101, *L. paracasei* LPC 1082, and *L. paracasei* LPC 1114) were further investigated for their antiadhesive properties against the two tested pathogens through CFU counts.

**Figure 2:**
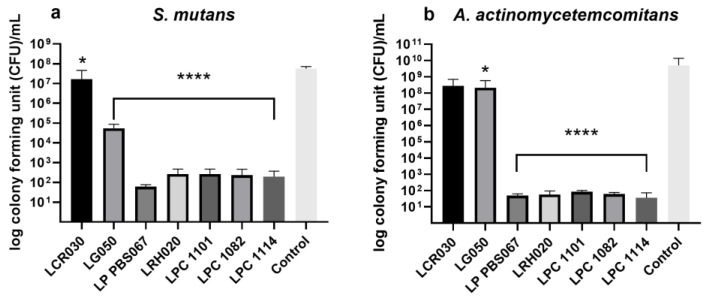
Pathogen colony forming unit (CFU) counts. Probiotic efficacy against (**a**) *S. mutans* and (**b**) *A. actinomycetemcomitans* adhesion and colonization. Results are expressed as mean ± SD of 3 independent experiments. * *p* < 0.05; **** *p* < 0.0001 vs. control (pristine medium only).

All probiotic strains reduced the number of pathogen cells able to adhere and subsequently replicate with respect to the control. *L. crispatus* LCR030 demonstrated a significant effect against *S. mutans* but not *A. actinomycetemcomitans*. Conversely, *L. gasseri* LG050 showed a significant inhibition against *A. actinomycetemcomitans* but not *S. mutans* (Figure 2).

*L. plantarum* PBS067, *L. rhamnosus* LRH020, *L. paracasei* LPC 1101, *L. paracasei* LPC 1082, and *L. paracasei* LPC 1114 induced a very significant decrease in cell adhesion of both *S. mutans* and *A. actinomycetemcomitans* and, as a consequence, a reduction in the CFU count (*p* < 0.0001; Figure 2a,b, respectively).

**Figure 3:**
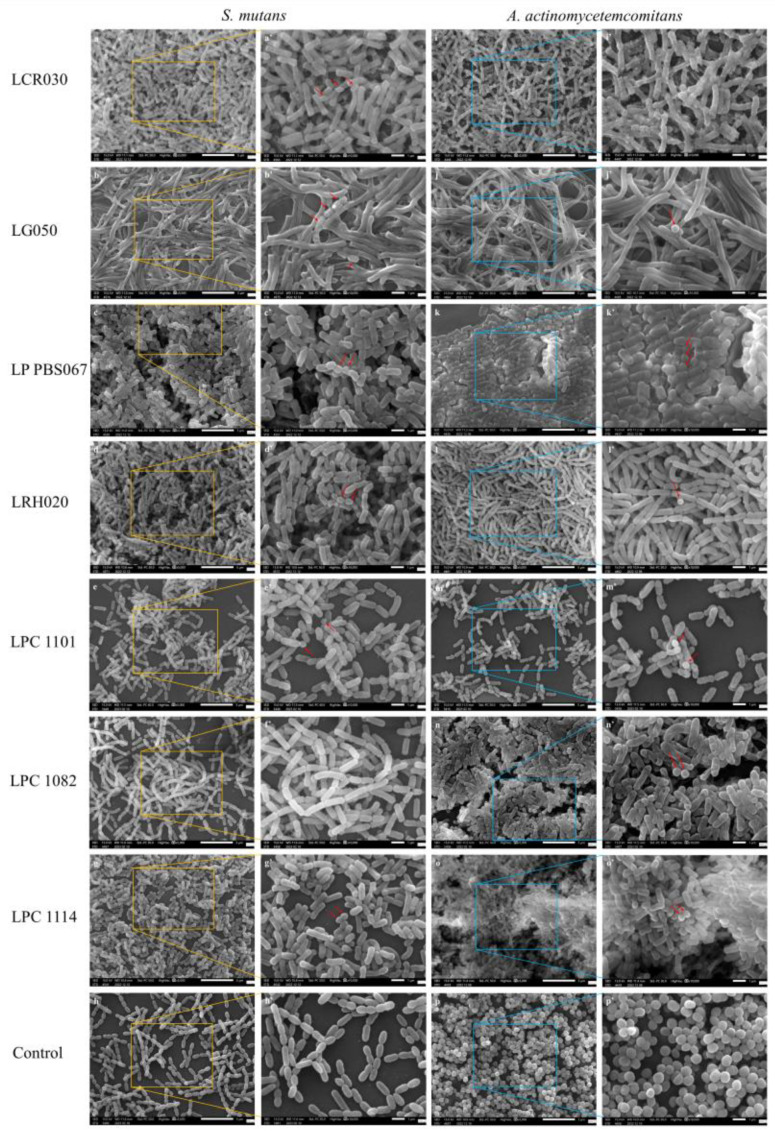
Representative images of probiotic efficacy in reducing pathogen adhesion. Probiotic inhibition of *S. mutans* and *A. actinomycetemcomitans* growth and adhesion at magnification 5000× ((**a**–**h**); (**i**–**p**)) and 10,000× ((**a’**–**h’**); (**i’**–**p’**)), respectively. The images were acquired by using a scanning electron microscope (SEM). The red arrows indicate the pathogen cells that were present. Yellow squares indicate the magnificated areas for *S. mutans*, blue squares for *A. actinomycetemcomitans.* LCR030: *L. crispatus* LCR030; LG050: *L. gasseri* LG050; LP PBS067: *L. plantarum* PBS067; LRH020: *L. rhamnosus* LRH020; LPC 1101: *L. paracasei* LPC 1101; LPC 1082: *L. paracasei* LPC 1082; LPC 1114: *L. paracasei* LPC 1114. Scale bars of 5 µm and 1 µm were shown in 5000× and 10,000× images, respectively.

The results described above were further confirmed by SEM images, which highlighted that the pathogenic bacteria were hindered from adhering to the probiotic layer formed during the pre-incubation time, resulting in a nearly complete prevention of colonization (Figure 3).

## 4. Discussion

Probiotics are non-pathogenic live and Generally Recognized as Safe (GRAS) microorganisms, mainly bacteria, which can benefit the hosts’ health if given in an appropriate amount [28]. Although the role of probiotics in the oral cavity has only recently been hypothesized and thus is still under-researched, the international literature already suggests that their use can be considered beneficial, given their ability to limit the development of pathogenic colonies [48].

We consider two pivotal periodontal pathobionts, *S. mutans* and *A. actinomycetemcomitans*, which trigger dental caries and periodontitis. Indeed, it is widely recognized that *S. mutans* adhere to the tooth enamel and use extracellular polysaccharides (EPSs) to build biofilms, resulting in dental caries consequently [10,49]. *A. actinomycetemcomitans* can contribute to infections of the periodontal tissue and harm the alveolar bone and periodontal ligaments, determining periodontal disease [49,50].

In addition to creating oral discomforts at the site of bacterial colonization, periodontal infections also affect the immune system systemically. In fact, a number of diseases in anatomically distant organs have been connected to periodontal infections [51]. It has been demonstrated that bacteria belonging to the oral cavity of subjects affected by periodontitis can disseminate through the vascular system, sustaining the inflammatory process by cytokine release and participating in cardiovascular diseases, atherosclerotic lesion onset or progression, neuroinflammation, and diabetes [52,53,54,55]. *A. actinomycetemcomitans* is recognized as a bacterial initiator of autoimmunity in rheumatoid arthritis (RA), together with *Porphyromonas gingivalis* [44,56]. Moreover, the damaged oral mucosal barrier resulting from periodontal disease can lead to the translocation of citrullinated oral bacteria into the circulatory system. This event activates both innate and adaptive immune responses, thereby contributing to the pathogenesis of RA [44,57].

In this context, the present study aims to evaluate the antibacterial and antiadhesive properties of a wide range of probiotic strains against *S. mutans* and *A. actinomycetemcomitans*. They may exert an effect by competitive exclusion of pathogen microorganisms, inhibition of their growth and expression of virulence factors, and production of antimicrobial substances [58]. These specific probiotic strains were chosen since no evidence about their potential beneficial effect in the oral context was demonstrated to the best of our knowledge. A successful method to screen various probiotic strains for bacteriocin-mediated competitive interactions is to use the overlay assay [45]. To determine the strain-specific activity, we performed the first screening on ten probiotic strains to detect antibacterial compound production. In the preventive model, the formation of the probiotic colonies and accumulation of the probiotic metabolites was allowed by a pre-incubation of 48 h with respect to the infection, while in the treatment model, a simultaneous incubation of the probiotic and pathogen strains was performed. The results demonstrated all the tested LAB have a positive effect in the containment of pathogen growth but only some of them induce a substantial reduction in this parameter; furthermore, the preventive model activity resulted to be more effective than the treatment assay. The inhibition zone measurement was conducted to investigate the inhibitory properties of different probiotics against *A. actinomycetemcomitans* ATCC 43718 serotype b in the work by Sulistiowati et al., confirming a strain-specific activity [43]. As highlighted by a recent review, probiotic bacteria may stimulate an immune response against pathogens, inhibiting the growth of bacteria that cause dental cavities and periodontal diseases and preventing inflammation and damage to tissues in the oral cavity [59].

Following this first screening selection, a series of seven strains were tested to understand their antiadhesive properties with respect to *S. mutans* and *A. actinomycetemcomitans*. *L. crispatus* LCR030 and *L. gasseri* LG050 confirmed their efficacy against *S. mutans* and *A. actinomycetemcomitans*, respectively. *L. plantarum* PBS067, *L. rhamnosus* LRH020, *L. paracasei* LPC 1101, *L. paracasei* LPC 1082, and *L. paracasei* LPC 1114 induced a very significant decrease in cell count and adhesion of both pathogens. LAB have a greater adherence to tissues and can compete with pathogens for growth factors, nutrition, and adhesion surfaces. This phenomenon happens not only in the intestinal mucosa but also in the oral cavity, where after adhering, probiotics group together and produce antimicrobial substances such as acids, bacteriocins, and peroxides, which prevent pathogenic bacteria from adhering and forming a biofilm [34,59,60]. Recently, several researches have been interested in the probiotic role in the prevention or eradication of bacterial biofilm on medical devices (i.e., different implants, artificial veins, or catheters). The competitive behavior of probiotics and/or their metabolites indeed hinders the pathogen attachment to medical device surfaces and inhibits their adhesion and aggregation; they also express disruptive properties against the pre-formed pathogen biofilms [61,62,63]. It is worth mentioning that LAB exert strain-specific probiotic properties. For example, the antimicrobial and antiadhesive properties of *L. plantarum* PBS067 and *L. rhamnosus* LRH020 were already demonstrated in the context of urogenital infections in human bladder epithelia, where the probiotics were able to co-aggregate with urogenital pathogens, limiting the colonization [64].

The use of probiotics as adjuvant treatment in different diseases seems promising. Several clinical trials reported improvement in halitosis, oral health, and related quality of life in subjects supplemented with different formulation of probiotics [65,66,67,68,69]. Moreover, many beneficial effects have been already demonstrated in the rebalance of the gut microbiota and also systemically [70,71,72]. Diverse *Lactobacillus* species exhibit immunomodulatory activities in the host gut, including enhanced antioxidant status and production of adhesion molecules and prevention of the establishment of neutrophil extracellular traps [73,74]. Gout and hyperuricemia, osteoarthritis, juvenile idiopathic arthritis, RA, osteoporosis and osteopenia, psoriasis, and spondyloarthritis may all be improved by probiotic supplementation [75]. An osteoarthritis rat model showed decreased pain severity, inflammation, and cartilage destruction when treated with *L. rhamnosus* [76].

## 5. Conclusions

The effects of specific probiotics can be considered as promising interventions to limit the oral pathogen growth within the oral cavity, thereby reducing their potential dissemination to distant anatomical niches. The antiadhesive and anti-biofilm properties exhibited by selected probiotic strains, such as *L. plantarum* PBS067, *L. rhamnosus* LRH020, *L. paracasei* LPC 1101, *L. paracasei* LPC 1082, and *L. paracasei* LPC 1114, can be considered crucial to set the basis for further in vitro investigation to better clarify their mechanism of action and their antimicrobial activity against other oral pathobionts. However, in vivo investigations are necessary to confirm these activities in predisposed patients.

In a future perspective, probiotic supplementation could be a complementary and alternative approach to classical medications to guarantee not only oral but also systemic health.

## Figures and Tables

**Table 1:** Measurements of inhibition halos of the probiotic strains against the two oral pathogens tested in both the preventive and treatment experiments. The diameter of the inhibition zone was classified as follow: 0 mm = no zone; diameter range: 1–15 mm = +; 16–30 mm = ++; 31–45 mm = +++; > 45 mm = ++++. Control: pristine acidic MRS broth (pH 4.5).

Probiotic Strains	*S. mutans*		*A. actinomycetemcomitans*	
	Model		Model	
	Preventive	Treatment	Preventive	Treatment
*L. acidophilus* PBS066	+++	++	++	++
*L. crispatus* LCR030	++++	++	+++	++
*L. gasseri* LG050	++	+	++++	+++
*L. plantarum* PBS067	+++	++	++++	+++
*L. reuteri* PBS072	++	++	+	++
*L. rhamnosus* LRH020	+++	++	+++	++
*B. lactis* BL050	+++	+	+++	+
*L. paracasei* LPC 1101	++++	++	+++	++
*L. paracasei* LPC 1082	+++	++	+++	++
*L. paracasei* LPC 1114	+++	++	++++	++
Control	No zone	No zone	No zone	No zone

## Data Availability

The original contributions presented in this study are included in this article; further inquiries can be directed to the corresponding author.
